# Editorial: The integration of the technology in clinical settings among neurological populations

**DOI:** 10.3389/fpsyg.2023.1145982

**Published:** 2023-05-11

**Authors:** Fabrizio Stasolla, Sara Bernini, Sara Bottiroli, Yiannis Koumpouros, Tanu Wadhera, Khalida Akbar

**Affiliations:** ^1^Giustino Fortunato University of Benevento, Benevento, Italy; ^2^Headache Science and Neurorehabilitation Centre, IRCCS Mondino Foundation, Pavia, Italy; ^3^Department of Public and Community Health, University of West Attica, Athens, Greece; ^4^Indian Institute of Information Technology (IIITU) Una, Una, Himachal Pradesh, India; ^5^Durban University of Technology, Durban, South Africa

**Keywords:** neurological impairments, clinical settings, technology, assessment, rehabilitation

Neurological populations commonly include any person who has a damage in the brain. Beside it, muscles, peripherical nerves, and spinal cord injury are broadly encompassed. Patients with neurological disorders due to either an acquired or a congenital brain injury may have different difficulties such as extensive motor impairments, cognitive deficits, sensorial disabilities, and lack of speech. Children and adolescents with cerebral palsy, individuals with autism spectrum disorders, adults with Alzheimer's or Parkinson's diseases, post-coma patients or stroke are targeted (Allen et al., 2020; Carod-Artal and García-Moncó, 2021). A common characteristic of the categories above detailed are their multiple disabilities which may range between mild-moderate and severe to profound depending on their different injuries. Consequently, patients with neurological disorders necessarily need professionals and families' support with an increased burden. As a result of their chronic diseases, neurological populations may suffer of a hampering situation. Whenever an injury occurs, it may cause relevant problems on their quality of life. That is, it may significantly compromise their image (Voinescu et al., 2021). To tackle this condition, one may envisage the integration of the technology (TBP) as a valid support to conventional clinical approaches (Yang et al., 2020).

Beside assistive technology, which may minimalize the distance between the behavioral repertoire and the environmental requests, new technologies can promote the use of augmented and/or virtual setups, to ensure the behavioral tracking, the experimental control, and the ecological validity for both assessment and rehabilitative purposes (Matamala-Gomez et al., 2019, 2021a; Stasolla et al., 2021). Serious games may offer funny and educational environments similar to the real life (Zucchella et al., 2014; Bottiroli et al., 2017, 2021). Telerehabilitation may enable a remote supervision either in a synchronous or asynchronous way (Quaglini et al., 2019; Bernini et al., 2021a,b). The integration of the technology in clinical settings may represent a valuable resource to enhance the healthcare and the quality of life among neurological populations (Matamala-Gomez et al., 2021b; Stasolla et al., 2021, 2022). A combined technology with assessment and rehabilitative objectives may additionally provide a comprehensive framework (Zhang et al., 2022).

The current Research Topic entitled “*The Integration of the Technology in Clinical Settings among Neurological Populations*” includes four high-quality contributions that present a relevant overview on the technological solutions available implemented in clinical settings for the assessment and recovery of patients with neurological conditions.

Karlsson et al. carried out a protocol study to evaluating the psychometric properties of a standardized cognitive assessment device that has been adapted for implementation with either a switch equipment or eye-gaze control technologies to be used in typically developing children or in case of motor and speech impairments. The authors also described a two-phase validation and acceptability investigation. This study protocol highlighted how technology should be used to avoid accessibility barriers and offer children with cerebral palsy updated evaluations. In this way, it is possible to support an overview of their cognitive and receptive language advantages and deficits.

Wandin et al. in a case report investigated a trained communication partner's use of responsive approaches in interactions of adults with Rett syndrome. A therapist with an expertise in responsive partner approaches and supported communication, interacted during 14 sessions with each of three participants. A gaze-controlled instrument and response approaches were adopted during all sessions. The Responsive Alternative and Augmentative Communication Style Scale (RAACS) was administrated to evaluate the communicative partner's responsive approaches through video-recorded sessions. Results indicated that the communication partner's implementation of responsive and scaffolding approaches is not a fixed construct but ranges in interactions with different non-speaking individuals as well as in different contexts. Hence, knowledge of the interaction patterns for the individual dyad could be adopted when designing a program aimed at improving the communication partner's use of responsive approaches.

Choong et al. explored the experience and thoughts of seven practicing therapists on the effect of Tele physical/physiotherapy therapy (TelePT), in children with chronic conditions during COVID-19 shutdowns. Authors highlighted that therapy implemented in the clinic did not impact the child's home context, suggesting the need of re-thinking on the design and implementation of the home exercise program. Thus, TelePT is considered a helpful supplement to standard, in-clinic PT practice. Specifically, they provided an evidence-based support on the ecological validity of how a child's therapy intervention is currently being conducted in the day-to-day life. TelePT could provide an excellent framework for emphasizing the relationship-driven, family-centered model into practice, scaffolding both professional skills development and increasing parental/family empowerment as highly effective partners in achieving therapy goals for the child.

Lancioni et al. evaluated a new technology-aided intervention focused on enhancing leisure, communication, and daily opportunities in four participants with intellectual and multiple disabilities. The program used a smartphone or tablet connected via Bluetooth to a two-switch-device. The device was useful to select leisure and communication stimuli and to manage the smartphone or tablet's delivery of step instructions for the activities scheduled. During the training all participants independently controlled the technology to access leisure events, made telephone calls, and achieved activities. Data suggested that the intervention could be a helpful support for helping people with intellectual and multiple disabilities to enhance their clinical condition in basic areas of daily life.

Taken together, the four studies of this Research Topic aimed to provide an overview about the possibilities of new technologies in clinical settings when applied to neurological patients. We hope that the contributions of this Research Topic can emphasize how to integrate such new technologies for the assessment and treatment of these patients in clinical settings.

## Author contributions

FS drafted the manuscript. SBo and SBe critically revised the manuscript for important intellectual content. YK, TW, and KA edited the manuscript. All authors conceived and designed the manuscript and approved the final version of the manuscript.
